# Afforestation does not increase the temperature sensitivity of soil organic carbon decomposition in the water-level fluctuation zone

**DOI:** 10.3389/fpls.2026.1914373

**Published:** 2026-08-05

**Authors:** Yan Lei, Wenling Zhang, Juguo Zhao, Haokun Shuai, Jiyang Lou, Xiaoye Fan, Tongyu Xu, Haonan Dong, Amit Kumar, Zhiguo Yu, Hepeng Li, Junjie Lin

**Affiliations:** 1 School of Materials and Environmental Engineering, Chengdu Technological University, Chengdu, China; 2 Department of Agricultural and Forestry Science and Technology, Chongqing Three Gorges Vocational College, Chongqing, China; 3 Zhejiang Guangchuan Engineering Consulting Co. Ltd., Hangzhou, China; 4 Hangzhou Huanzheng Environmental Technology Limited Company, Hangzhou, China; 5 Lishui Center for Soil and Solid Waste Management, Lishui, China; 6 Liandu Bureau of Agriculture and Rural Affairs, Lishui, China; 7 School of Environment and Natural Resources, Zhejiang University of Science and Technology, Hangzhou, China; 8 School of Hydrology and Water Resources, Nanjing University of Information Science and Technology, Nanjing, China; 9 Zhejiang Academy of Forestry, Hangzhou, China

**Keywords:** climate change, flood-tolerant tree, mineral-associated organic carbon, soil organic carbon, temperature sensitivity, Three Gorges Reservoir

## Abstract

Taxodium “Zhongshanshan”, a flood-tolerant tree species, was primarily applied for afforestation restoration practice of soil erosion control in the water-level fluctuation (WLF) zone. However, how afforestation effects influence the temperature sensitivity (Q_10_) of soil organic carbon (SOC) mineralization remains poorly understood in the WLF zone under climate warming. Here, we collected soils from paired planted and unplanted plots in the WLF zone and non-flooding zone in the Three Gorges Reservoir after six years of Taxodium “Zhongshanshan” plantation. The Q_10_ was estimated during a 60-day incubation at 20 °C and 30 °C. Soil carbon/nitrogen fractions, potential of minerals to adsorb SOC, and SOC molecular structure were analysed at the end of the incubation. The afforestation increased cumulative SOC mineralization by 53.2% in the non-flooding zone but reduced it by 13.7% in the WLF zone over the 60-day incubation. The afforestation increased Q_10_ by 31.3% in the non-flooding zone but did not change Q_10_ in the WLF zone. The six-year plantation elevated nitrate nitrogen and dissolved total nitrogen in the WLF zone, yet reduced them in the non-flooding zone. The afforestation decreased the SOC stability in the WLF zone in contrast to the non-flooding zone. Correspondingly, the increase in the potential of minerals to adsorb SOC, indicated by the molar ratios of metals to SOC, i.e., molar (Fe_d_ + Al_d_)/SOC, molar (Fe_o_ + Al_o_)/SOC, and exchangeable Ca-to-SOC (Ca_exe_/SOC), was greater in the WLF zone than in the non-flooding zone. Collectively, afforestation-induced shifts in SOC stability, nutrient supply, and mineral protection jointly mitigate the Q_10_ of SOC mineralization in the WLF zone, laying a critical theoretical foundation for wetland carbon sequestration restoration via flood-tolerant tree species.

## 1 Introduction

Soil organic carbon (SOC) is the largest active carbon reservoir in terrestrial ecosystems, storing 1500–2400 Pg C globally, constituting 2–3 times the atmospheric and vegetation carbon pools (Jobbágy and Jackson, 2000). A minor disturbance in SOC storage capacity may profoundly shift the concentrations of atmospheric carbon dioxide (CO_2_) (Weng et al., 2012). Future carbon-climate feedback risks are severe due to warming-triggered terrestrial carbon loss and nonlinear land carbon responses (Fang et al., 2026). The long-term dynamics of SOC decomposition are jointly regulated by land-use transformation and climate change, representing a central research focus in global terrestrial carbon cycling (Xu et al., 2026). It is a critical pathway to render soil carbon sequestration by maintaining soil carbon stability for mitigating climate change and achieving carbon neutrality targets (Lal, 2018; Lan et al., 2025).

Rapid expansion in forest cover contributed to 44% of the terrestrial carbon sink in China (Yu et al., 2022). However, the afforestation effects are context-dependent, which is beneficial for boosting SOC density in carbon-poor soils rather than carbon-rich soils (Hong et al., 2020). Afforestation increases SOC sequestration by accumulating fungal residual carbon indicated by amino sugars in a seasonally flooded marshland (Tang et al., 2024). Inconsistent SOC responses have been reported following afforestation, including increases, decreases, or no change in carbon stocks, which are modulated by tree species, stand age, soil properties, and regional climate (Hou et al., 2020; Yu et al., 2025). Thus, the effects of afforestation on SOC sequestration remain highly uncertain (Amoakwah et al., 2022). To deeply understand how tree plantation expansion affects the terrestrial carbon budget is critical for reliable terrestrial carbon accounting and climate policy design (Fagan et al., 2022).

The water-level fluctuation (WLF) zone is a typical aquatic–terrestrial ecotone characterized by periodic flooding and drying cycles, with fragile and sensitive ecosystem functions (Bao et al., 2015; Kumar et al., 2024). Seasonal variation in water level results in a decrease in vegetation species diversity in the WLF zone (Zhu et al., 2020). Such drastic hydrological alterations cause severe vegetation degradation, soil erosion, and SOC depletion, profoundly altering regional carbon cycling (Mandal and Banik, 2025). Although wetland ecosystems account for 10% of the terrestrial carbon pool and possess strong carbon sequestration potential, how periodic water-level fluctuations modulate SOC mineralization and its temperature sensitivity (Q_10_) remains poorly understood (Dong et al., 2021; Zhang et al., 2020b).

Global climate change has intensified substantially, with a global mean temperature increase of 0.85 °C recorded from 1880 to 2012 and a potential warming of up to 4.8 °C projected by the end of this century without effective mitigation (Masson-Delmotte et al., 2021). Soil carbon mineralization increases exponentially with rising temperature, and the Q_10_ coefficient is a critical parameter for characterizing the positive feedback between soil carbon release and climate warming (Lin et al., 2025b). However, the current model assumes a constant Q_10_ value and fails to account for fluctuations in hydrological conditions, which introduces substantial uncertainties in predictions of carbon-climate feedbacks (Wang et al., 2014; Zhou et al., 2023).

Afforestation alters vegetation productivity, litter input, and soil microenvironments (e.g., temperature, moisture, and pH), further reshaping soil microbial community structure and enzyme activities and regulating the responses of SOC mineralization to warming (Lin et al., 2023; Zhang et al., 2021). However, most existing studies focus on upland ecosystems, while the WLF zone in the big reservoir remains understudied (He et al., 2024). Further, how afforestation modulates Q_10_ via altering soil physicochemical properties, nutrient availability, and microbial processes in the reservoir WLF zone remains unclear (Chen et al., 2024; Qiu et al., 2023). In particular, the mechanisms underlying SOC stabilization and Q_10_ regulation following flood-tolerant tree afforestation in the WLF zone lack systematic empirical evidence.

To investigate the effects of afforestation and hydrological regime on SOC mineralization and Q_10,_ soils collected from paired 6-year planted and adjacent unplanted sites in the WLF zone and non-flooding zone were incubated for 60 days at 20 °C and 30 °C to quantify the responses of SOC mineralization to warming. In addition, soil physicochemical properties, carbon and nitrogen fractions, the potential of minerals to adsorb SOC, and microbial-related processes were measured to clarify the underlying variations in SOC mineralization and Q_10_. The study aimed to improve our understanding of soil carbon cycle responses to vegetation restoration and climate warming in reservoir WLF ecosystems and provide a scientific basis for carbon-oriented ecological restoration and management of reservoir WLF ecosystems.

## 2 Materials and methods

### 2.1 Site description

The hydrological regulation regime forms a 350 km^2^ WLF zone in the Three Gorges Reservoir (Willison et al., 2013). *Taxodium* “Zhongshanshan” is a widely applied flood-tolerant hybrid tree species for ecological restoration in the WLF zone of the Three Gorges Reservoir region. It exhibits strong adaptability to long-term submergence, rapid growth, and high tolerance to saline-alkali conditions, making it the dominant afforestation species for WLF zone restoration and soil erosion control. The paired sites with similar topography, soil parent material, and hydrological conditions were selected, including planted and unplanted plots. The plantation had uniform stand age and planting density. The unplanted site was free of planting and human disturbance. The planted and unplanted plots were arranged adjacently to minimize spatial heterogeneity, with a 5 m buffer zone separating each plot pair. The study sites were located at 30°50’-30°52’ N, 108°28’-108°29’E with a subtropical humid monsoon climate (MAT = 18.0°C, MAP = 1181.2 mm) and purple soils. Taxodium “Zhongshanshan” seedlings were planted at a 3 m × 3 m spacing, yielding average tree heights of 3.2–3.8 m and average diameter at breast height of 4.2–5.1 cm across elevational gradients.

### 2.2 Soil sampling

Field sampling was performed at the annual low water level in July 2024 after six years of artificial Taxodium “Zhongshanshan” restoration in the WLF zone of the Three Gorges Reservoir in Wanzhou, Chongqing, China. Two typical hydrological elevations (meters mean sea level; m asl) were set at the sites of the WLF zone at 165 m asl and the non-flooding zone at 180 m asl. In total, sixteen 20 m × 10 m plots were arranged under two water level elevations and two planting treatments with four replicates. Three random soil cores were taken from 0–20 cm by plum blossom sampling in each plot and combined as a single composite sample. In total, 16 composite soils were collected, including 2 plantation treatments × 2 hydrological regimes × 4 plots. All samples were air-dried and sieved through a 2 mm mesh. Soil physicochemical properties were determined after removing visible plant residues, animal debris, and rock fragments (**Table 1**).

**Table 1:** Soil properties in the water level fluctuation zone and non-flooding zone.

Items	NF		WLF	
	Unplanted	Planted	Unplanted	Planted
SOC (%)	1.79 ± 0.08a	0.44 ± 0.11b	1.02 ± 0.08a	0.81 ± 0.07b
TN(%)	0.067 ± 0.001a	0.031 ± 0.001b	0.045 ± 0.002a	0.044 ± 0.001a
C/N	26.75 ± 1.54a	13.89 ± 3.74b	22.42 ± 1.29a	18.65 ± 1.66b
pH	7.72 ± 0.33a	7.75 ± 0.21a	7.41 ± 0.19a	7.56 ± 0.27a
WHC(%)	31.93 ± 0.41a	26.91 ± 0.69b	34.80 ± 2.26b	42.73 ± 1.74a

Values are the mean ± standard error (n=4). Different lowercase letters indicate significant differences between planted and unplanted treatments at each region at *p* < 0.05. WLF, water level fluctuation zone; NF, non-flooding zone.

### 2.3 Soil incubation

20 g of dry soil was pre-incubated at 20 °C with 40% of soil water-holding capacity (WHC) to restore microbial activity for 7 days. After the pre-incubation, samples were formally incubated at 20 °C and 30 °C with 60% WHC for 60 days. Soil moisture was maintained by weighing. Soil CO_2_ emissions were captured using 1 M NaOH solution on days 3, 7, 14, 28, 35, 49, and 60 during incubation. The content of inorganic carbon in NaOH solution was quantified by HCl titration after the fixation of BaCl_2_ solution.

The temperature sensitivity (Q_10_) of SOC mineralization describes the relative increase in mineralization rate per 10 °C rise in temperature, serving as a core metric to quantify carbon cycle–climate feedback. The Q_10_ was calculated using Eq. 1 (Chen et al., 2010).

Q_10_ = (R_2_/R_1_)(10/(t2-t1))(1).

where R_2_ and R_1_ represent the cumulative soil CO_2_ emission rates over the whole incubation duration at 30 °C and 20 °C, respectively; t_2_ and t_1_ are the corresponding incubation temperatures of 30 °C and 20 °C.

### 2.4 Laboratory analysis

Soil pH and soil WHC were measured via a soil–water suspension (1:2.5, w/v) and a saturated drainage approach, respectively (Klute, 1986; Thomas, 1982). SOC was quantified using the potassium dichromate oxidation spectrophotometric method (Walkley and Black, 1934). Hot water-extractable organic carbon (HWEOC) was extracted via 70 °C water bathing and determined using a TOC analyzer (Ghani et al., 2003). Soil microbial biomass carbon (MBC) and nitrogen (MBN) were measured using the chloroform fumigation–extraction method, with a conversion factor of 0.45 for calculating microbial biomass (Vance et al., 1987). The extracts from unfumigated soils were used for dissolved organic carbon (DOC) measurement. Particulate organic carbon (POC) and mineral-associated organic carbon (MAOC) were fractionated via sodium hexametaphosphate dispersion and wet sieving, with their contents calculated by mass difference (Cotrufo et al., 2019; Poeplau et al., 2018). Total nitrogen (TN) and dissolved total nitrogen (DTN) were determined using the alkaline potassium persulfate digestion ultraviolet spectrophotometric method (Cabrera and Beare, 1993). Soil ammonium nitrogen (NH_4_^+^-N) and nitrate nitrogen (NO_3_^-^-N) were extracted with 2 M KCl solution and quantified via colorimetric and ultraviolet spectrophotometric assays, respectively (Helmke, 1996).

The potential of minerals to adsorb SOC (mineral protection) was quantified via the molar metal-to-SOC ratios derived from bulk soil measurements and SOC concentrations, including molar (Fe_d_ + Al_d_)/SOC, exchangeable Ca-to-SOC (Ca_exe_/SOC), and molar (Fe_o_ + Al_o_)/SOC (Gentsch et al., 2018). Exchangeable Ca (Ca_exe_), free Fe/Al oxides (Fe_d_+Al_d_), and amorphous Fe/Al oxides (Fe_o_+Al_o_) were extracted using ammonium acetate, citrate–bicarbonate–dithionite, and acid ammonium oxalate methods, respectively, and their concentrations were determined by ICP-OES (Thomas, 1982). Soil organic carbon functional group characteristics were analyzed via Fourier transform infrared spectroscopy (FTIR). Relative peak areas of typical functional group bands (2930, 2850, 1635, 1430, and 1035 cm^-^¹) were integrated to calculate the SOC stability index (rA1635/rA2930) (Hou et al., 2021; Nestle et al., 2008).

### 2.5 Statistical analysis

Independent-sample t-tests were used to examine differences in soil properties between the planted and unplanted plots at the same elevation. A linear mixed model was applied to examine the effects of planting, flooding, and their interaction. A probability level of *P* < 0.05 was considered statistically significant. All statistical analyses were conducted using IBM SPSS Statistics 26.0, and all figures were plotted with Origin 2021 for data visualization.

## 3 Results

### 3.1 Effects of afforestation on SOC mineralization and Q_10_

**Figure 1:**
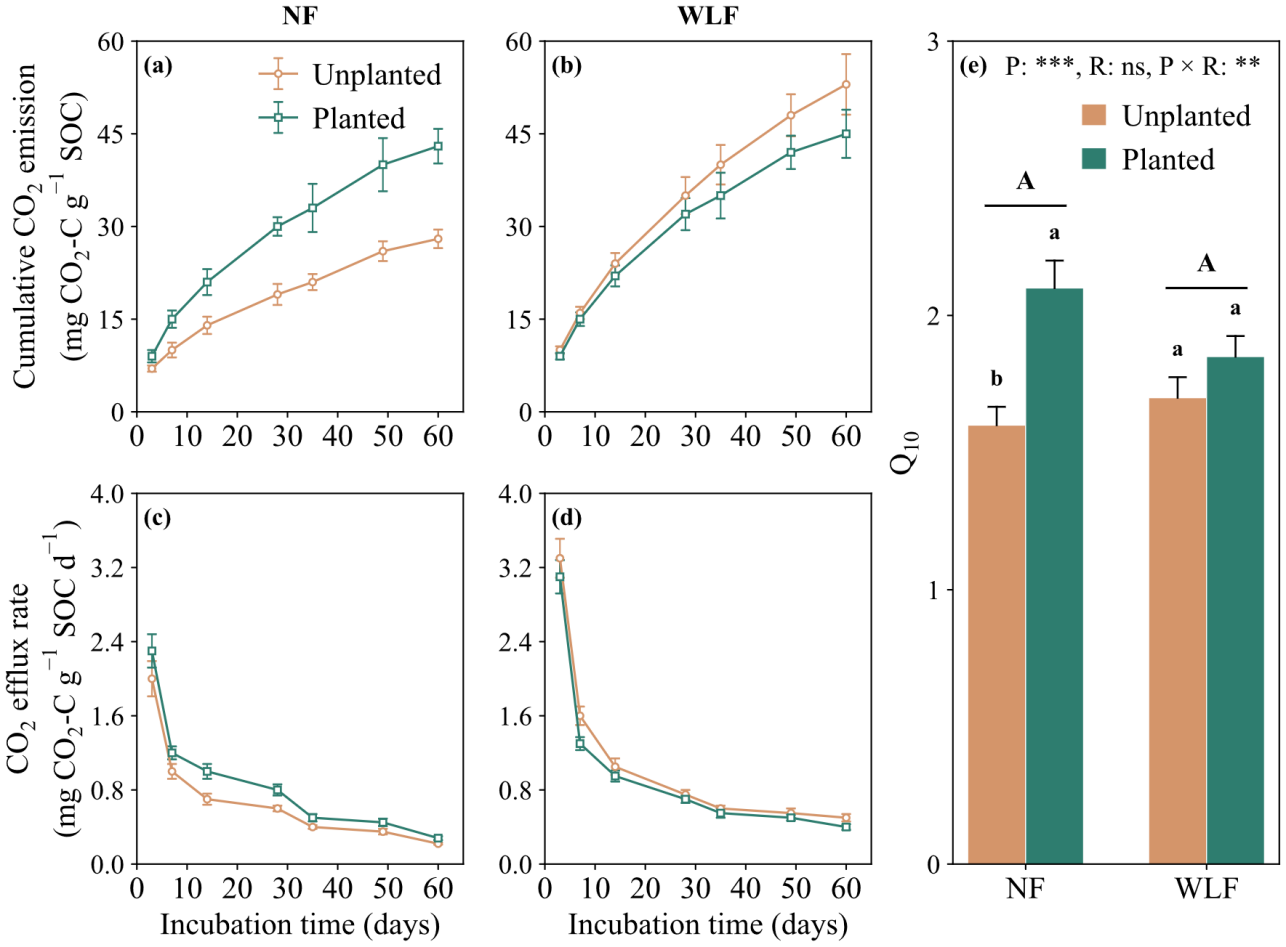
CO_2_ emissions (a-d) and their temperature sensitivity (Q_10_, e) from six-year planted and unplanted soils in the WLF zone and non-flooding zone over the 60-day incubation. Values are the mean ± standard error (n=4). Different lowercase letters indicate significant differences between planted and non-planted in each site, while different uppercase letters indicate significant differences between WLF and NF at *p* < 0.05. R, Regions (WLF vs NF); P, Plantations (Planted vs Unplanted). ***p < 0.001, **p < 0.01; ns, *p* > 0.05. WLF, WLF zone; NF, non-flooding zone.

In the non-flooding zone, planted plots exhibited higher SOC mineralization rates than unplanted plots (**Figure 1c**). Afforestation increased cumulative SOC mineralization by 34.1%, indicating a strong promotional effect on SOC decomposition (**Figure 1a**). In contrast, periodic flooding reversed this regulatory pattern in the WLF zone. The planted plots showed a lower range of SOC mineralization rates and a 15.0% reduction in cumulative mineralization compared with unplanted plots, reflecting an inhibitory effect of afforestation on SOC mineralization under water-level fluctuation conditions (**Figures 1b, d**). The Q_10_ of SOC mineralization ranged from 1.6 to 2.1 across all samples, falling within the typical range of Q_10_ for terrestrial SOC decomposition. Afforestation altered the Q_10_ in a hydrology-dependent manner. Under non-flooding conditions, afforestation increased Q_10_ by 31.3%, rising from 1.6 in unplanted sites to 2.1 in plantation sites (*p* < 0.05), demonstrating that afforestation strengthened the temperature dependence of SOC mineralization. At the WLF zone, the Q_10_ did not differ between planted and unplanted plots (**Figure 1e**).

### 3.2 Effects of afforestation on SOC and nitrogen fractions

**Figure 2:**
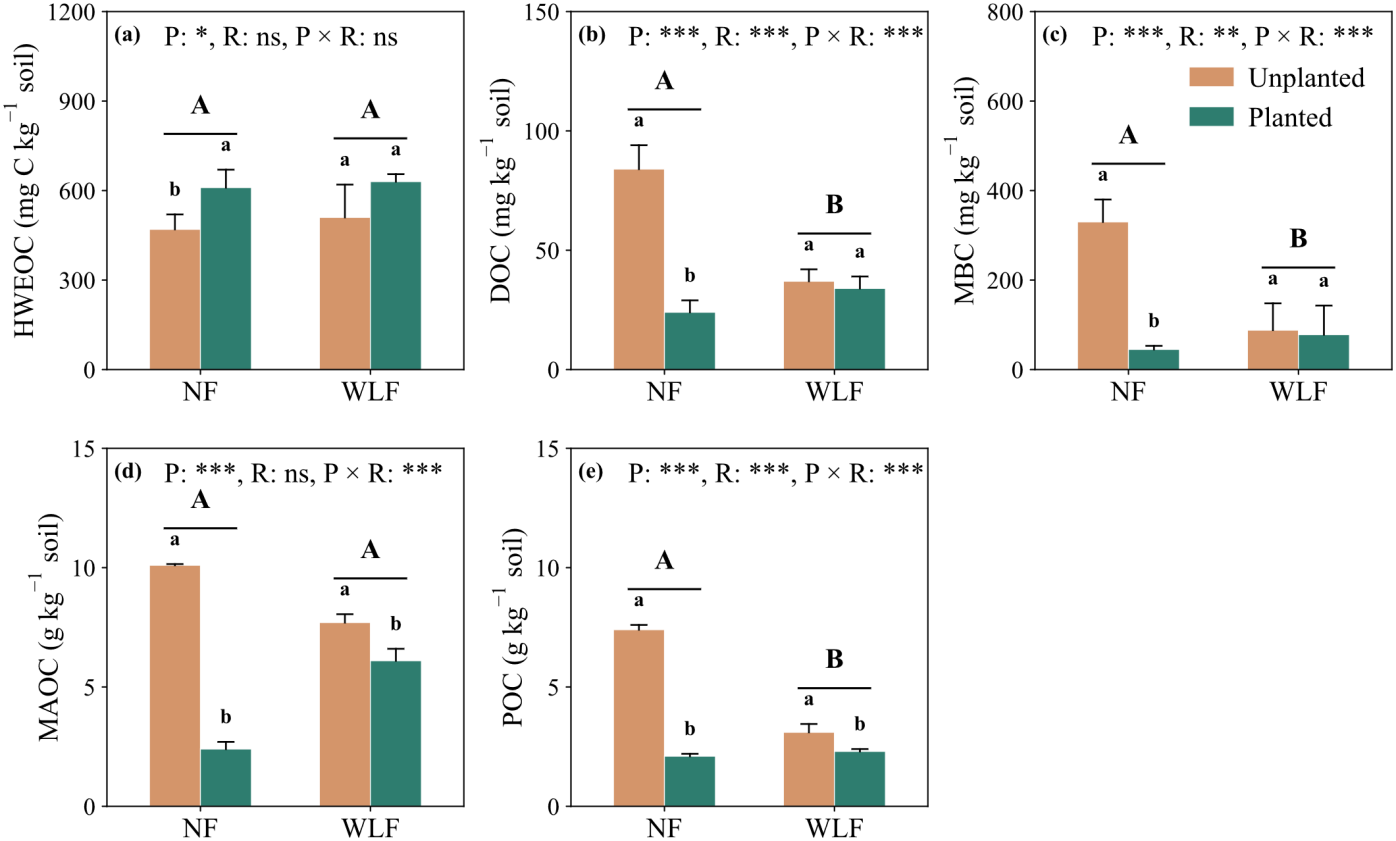
Soil carbon components in the WLF zone and non-flooding zone under planted and unplanted plots. WLF, WLF zone; NF, non-flooding zone; HWEOC, Hot-water-extractable organic carbon; DOC, dissolved organic carbon; MBC, microbial biomass carbon; MAOC, mineral-associated organic carbon; POC, particulate organic carbon. Values are the mean ± standard error (n=4). Different lowercase letters indicate significant differences between planting and non-planting in each region, while different uppercase letters indicate significant differences between WLF and NF at *p* < 0.05. R, Regions (WLF vs NF); P, Plantations (Planted vs Unplanted). ***p < 0.001; **p < 0.01; *p < 0.05; ns, *p* > 0.05.

**Figure 3:**
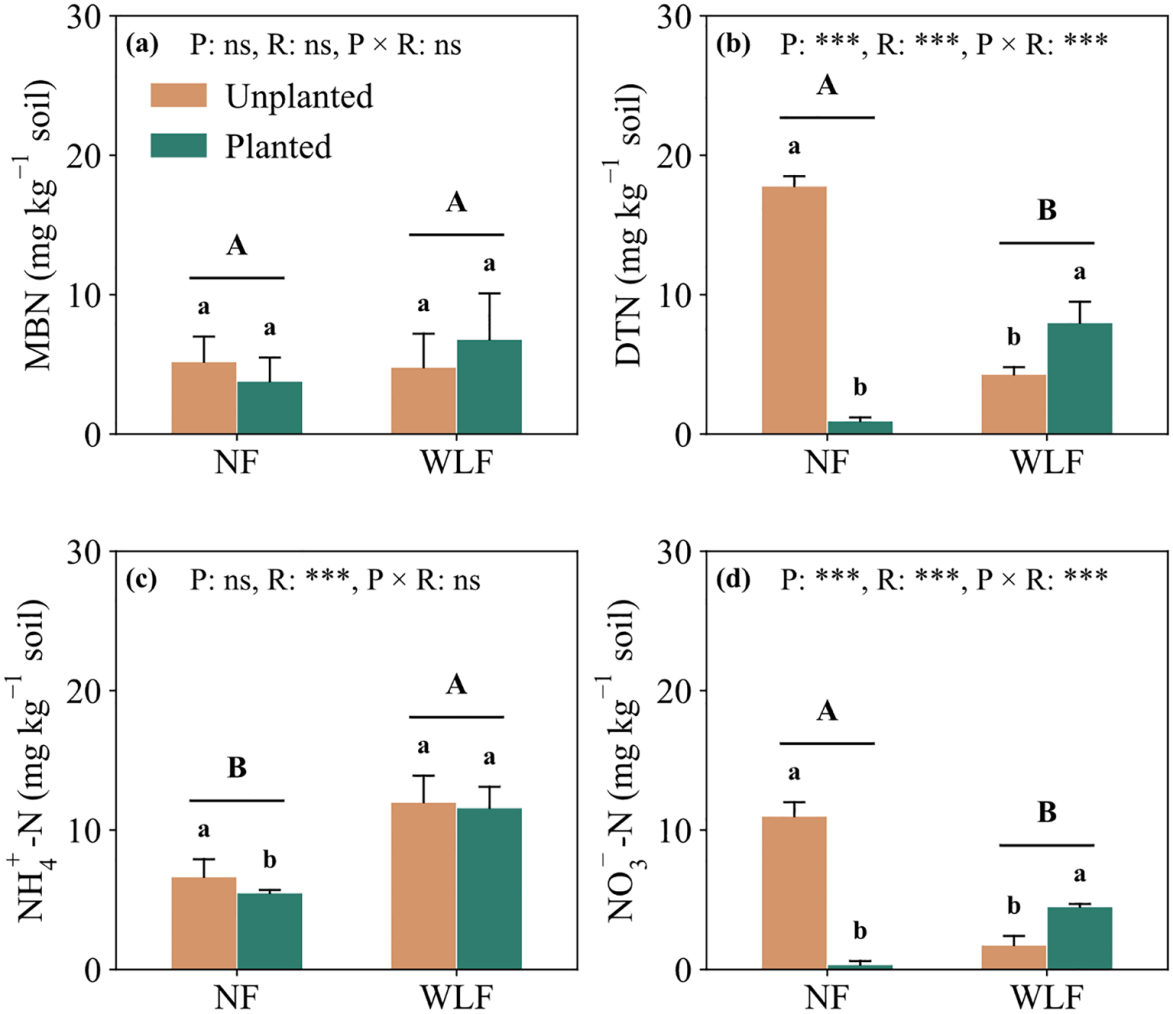
Soil nitrogen components in the WLF zone and non-flooding zone under planted and unplanted plots. WLF, WLF zone; NF, non-flooding zone; MBN, microbial biomass nitrogen; DTN, dissolved total nitrogen; NH_4_^+^-N, ammonium nitrogen; NO_3_^-^-N, nitrate nitrogen. Values are the mean ± standard error (n=4). Different lowercase letters indicate significant differences between planted and unplanted treatments at each region, while different uppercase letters indicate significant differences between WLF and NF at *p* < 0.05. R, Regions (WLF vs NF); P, Plantations (Planted vs Unplanted). ***p < 0.001; **p < 0.01; *p < 0.05; ns, *p* > 0.05.

Afforestation reduced SOC content in non-flooding and WLF zones (**Table 1**). In non-flooding zone, afforestation declines labile SOC fractions relative to unplanted plots, with DOC, MBC, and POC decreased by 71.8%, 86.6%, and 71.6%, respectively (**Figures 2b, c, e**), while the stable MAOC fraction was reduced by 76.2% (**Figure 2d**). By contrast, afforestation increased HWEOC content by 28.7% in non-flooding zone (**Figure 2a**). At the WLF zone, afforestation had no effects on HWEOC, DOC, and MBC (**Figures 2a–c**), whereas reduced POC and MAOC contents by 26.7% and 26.7%, respectively, compared with unplanted plots (**Figures 2d, e**). In the WLF zone, MBN and NH_4_^+^-N showed no differences between planted and unplanted plots (**Figures 3a, c**), whereas afforestation increased DTN and NO_3_^--^N contents by 78.3% and 161.1%, respectively (**Figures 3b, d**). Spatially, the non-flooding zone had higher DTN and NO_3_^--^N concentrations but lower NH_3_^+^-N concentrations than the WLF zone, with consistent MBN levels across both the WLF and non-flooding zones (**Figure 3**).

### 3.3 SOC structures and mineral protection

**Figure 4:**
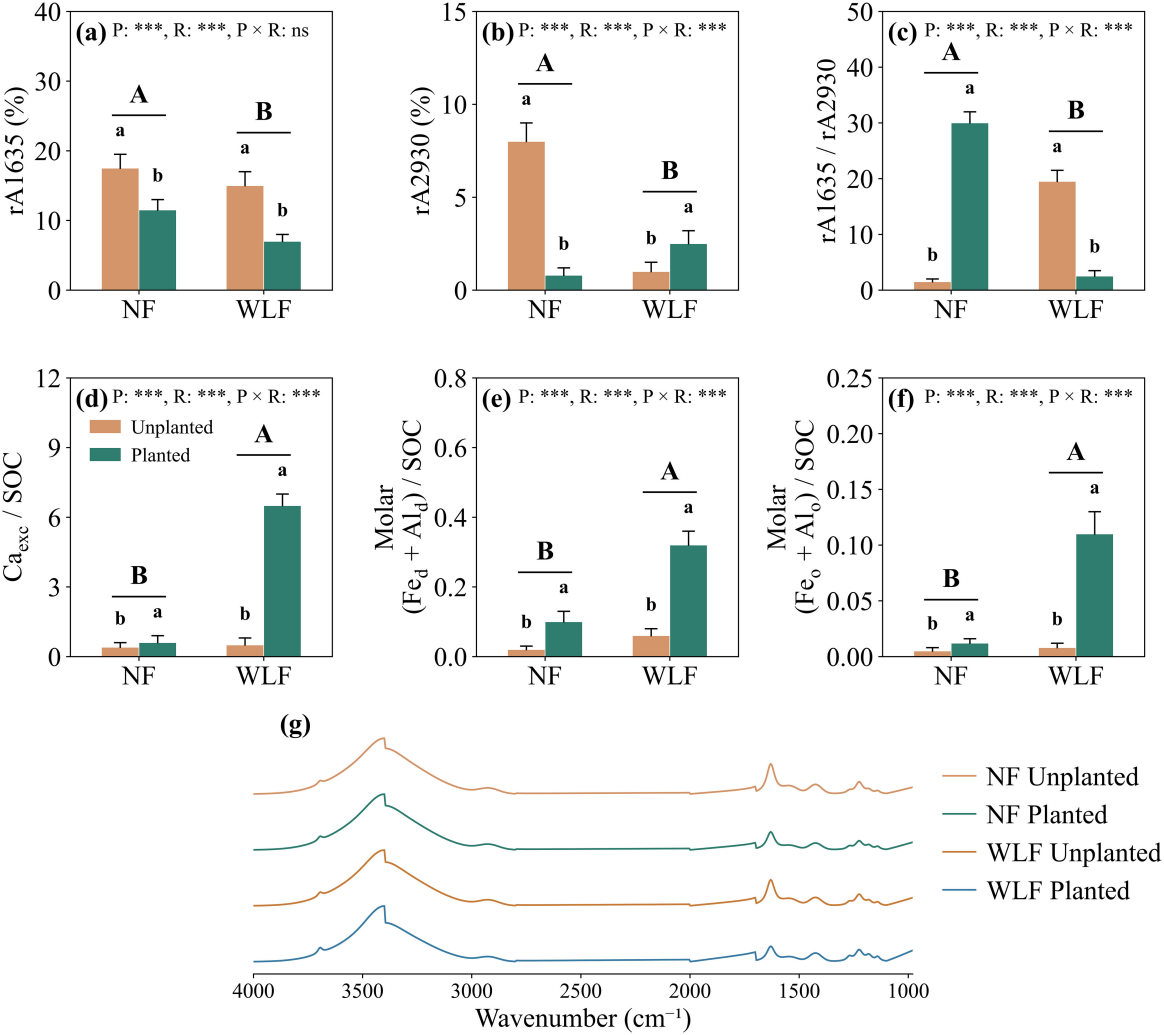
The SOC stability (a-c, g) and potential of minerals to adsorb SOC (molar (Fe_d_ + Al_d_)/SOC, exchangeable Ca-to-SOC (Ca_exe_/SOC), and molar (Fe_o_ + Al_o_)/SOC, d-f) in the WLF zone and non-flooding zone. WLF, WLF zone; NF, non-flooding zone; Values are the mean ± standard error (n=4). Different lowercase letters indicate significant differences between planted and unplanted treatments at each region, while different uppercase letters indicate significant differences between WLF and NF at *p* < 0.05. R, Regions (WLF vs NF); P, Plantations (Planted vs Unplanted). ***p < 0.001; **p < 0.01; *p < 0.05; ns, *p* > 0.05.

Afforestation increased the rA1635/rA2930 ratio, indicating improved molecular structural stability of SOC in the non-flooding zone (**Figures 4c, g**). In contrast, afforestation reduced the rA1635/rA2930 ratio in the WLF zone, suggesting weakened intrinsic molecular stability of SOC in periodically flooded habitats (**Figure 4c**, **Table 2**). In non-flooded conditions, afforestation increased the potential of minerals to adsorb SOC, indicated by the molar ratios of metals to SOC, i.e., molar (Fe_d_ + Al_d_)/SOC, exchangeable Ca-to-SOC (Ca_exe_/SOC), and molar (Fe_o_ + Al_o_)/SOC by 5.0, 1.5, and 3.5 times, respectively (**Figures 4d–f**). A more substantial enhancement occurred in the WLF zone, with the three molar ratios of metals increased by 5.1, 15.8, and 15.0 times in the planted plots compared with the unplanted plots (**Figures 4d–f**). Meanwhile, the potential of minerals to adsorb SOC was higher in the WLF zone than in the non-flooding zone, indicating that periodic water-level fluctuation further strengthened the physicochemical protection of SOC (**Figures 4d–f**).

**Table 2:** Organic carbon assignments, relative peak areas and peak wavenumbers in the FTIR spectra of planted and unplanted plots under WLF and non-flooding.

Wavenumber (cm^-1^)	Relative peak areas (%)				Band ranges (cm^–1^)	Organic carbon assignments
	NF		WLF			
	Unplanted	Planted	Unplanted	Planted		
2930	8.08 ± 0.28a	0.37 ± 0.03b	0.79 ± 0.04b	2.30 ± 0.22a	3010–2800	Aliphatic C–H stretch
2850	0.05 ± 0.00a	0.05 ± 0.08a	0.01 ± 0.00a	0.005 ± 0.00b	2855–2850	Aliphatic C–H symmetric stretch
1635	17.16 ± 1.76a	11.34 ± 0.24b	14.94 ± 1.69a	6.99 ± 0.61b	1660–1580	Carboxylate C=O stretch or Aromatic C=C stretch
1430	3.35 ± 0.23a	2.32 ± 0.23b	2.48 ± 0.13b	4.70 ± 0.43a	1450–1400	Primary amides C=N or C–O or C–H bend
1035	46.86 ± 1.20a	41.70 ± 0.89b	52.47 ± 1.89a	43.22 ± 1.00b	1160–1000	Carbohydrate Ester, phenol C–O stretch

Different lowercase letters denote significant differences between plant regimes within NF or WLF at *p* < 0.05. Values are the mean ± standard error (n = 4).

## 4 Discussion

### 4.1 Responses of SOC mineralization to afforestation

Soil moisture is a key factor regulating SOC mineralization (Curtin et al., 2012). The responses of SOC mineralization to afforestation showed strong vegetation–hydrology interactive effects between WLF zone and non-flooding zone (**Figures 1a–d**), highlighting that periodic water fluctuation reshapes plant-soil carbon feedback distinctively from permanent upland ecosystems (Ye et al., 2020). In the non-flooding zone, afforestation promoted cumulative SOC mineralization by 34.1% compared with unplanted plots (**Figure 1a**). The enhanced SOC decomposition was primarily attributed to improved microenvironmental conditions and increased substrate availability following vegetation restoration (Wang et al., 2025). Afforestation altered soil physical properties and activated microbial metabolism, thereby accelerating the SOC turnover (Zhang et al., 2020a). Relatively stable water conditions in non-flooding uplands sustain continuous microbial activity and facilitate SOC decomposition, consistent with the carbon availability-quality tradeoff framework that aerobic environments maximize microbial access to labile carbon substrates (Zhang et al., 2024a).

In contrast, periodic inundation strongly modulates soil carbon biogeochemical processes in reservoir WLF ecosystems (Wu et al., 2024). Afforestation suppressed cumulative SOC mineralization in the WLF zone by 15.0% (**Figure 1b**). Flooding alters soil redox conditions, restructures microbial communities, and limits the decomposition of labile SOC fractions (Cheng et al., 2025; McNicol and Silver, 2014). Although moderate soil moisture can stimulate microbial activity, frequent flooding–drying cycles in the WLF zone disrupt microbial stability and constrain continuous SOC mineralization (Zhang et al., 2026). Such hydrological disturbance creates a unique carbon preservation mechanism exclusive to WLF zones, where ferric oxides formed during dry phases re-adsorb newly produced DOC under submerged anoxia (Philben et al., 2024). The SOC mineralization rates were closely correlated with SOC, DOC, and MBC contents, indicating that the availability of labile SOC was the primary driver of SOC decomposition (Han and Wang, 2024). Additionally, positive correlations between mineralization rate and soil nitrogen components suggested that nitrogen availability co-regulated microbial decomposition processes in the WLF soils (Averill and Waring, 2018).

### 4.2 Effects of afforestation on Q_10_ of SOC mineralization

The Q_10_ of SOC mineralization ranged from 1.6 to 2.1 (**Figure 1e**), which falls within the universal range of 1.2–2.8 for terrestrial soils, indicating reliable and representative temperature response characteristics of SOC decomposition (Xu et al., 2026). This range aligns with regional investigation results that riparian soils have an average Q_10_ of 1.1-2.1, driven by the decomposability of SOC (Yu et al., 2025). Afforestation increased Q_10_ in the non-flooding zone, suggesting that plantation enhanced the temperature dependence of SOC mineralization under stable hydrological conditions (Li et al., 2022). Higher Q_10_ is generally attributed to improved substrate quality and elevated microbial metabolic potential from afforestation, which render SOC decomposition more sensitive to rising temperature, matching the core prediction of the carbon quality-temperature hypothesis (Davidson and Janssens, 2006; Pei et al., 2024). Under steady aerobic conditions, fresh root exudates and litter inputs raise the proportion of labile alkyl carbon fractions, whose decomposition carries higher activation energy and stronger temperature sensitivity (Erhagen et al., 2013).

However, afforestation had divergent effects on Q_10_ under flooding stress (He et al., 2024). In the WLF zone, the Q_10_ of SOC mineralization did not change after afforestation, which was closely linked to the unique substrate conditions and flooding-induced microbial constraints (Zhao et al., 2023). Flooding disturbance reduced the proportion of easily decomposable SOC fractions and weakened microbial response to temperature variation, thereby offsetting the promotional effect of plantation on Q_10_ (Shen et al., 2026; Yu et al., 2025). Alternating anoxic-aerobic cycles inhibit the accumulation of thermally sensitive labile carbon and stabilize MAOC pools that feature low temperature responsiveness (Qin et al., 2021). Such hydrology-dependent Q_10_ variations highlight that the climate–carbon feedback of reservoir drawdown soils is highly regulated by water-level fluctuation and vegetation restoration (Qiu et al., 2023; Zhang et al., 2026).

Nevertheless, the Q_10_ values in this study were determined via field sampling combined with laboratory temperature-controlled incubation. Laboratory homogenization eliminates *in-situ* vertical stratification of moisture, oxygen, and mineral fractions, leading to systematically higher Q_10_ estimates compared with long-term field *in-situ* monitoring. Thus, lab-derived Q_10_ can only represent the potential response capacity of SOC decomposition to rising temperature under standardized aerobic incubation conditions, and cannot capture seasonal inundation disturbance and vertical carbon loss along soil profiles in natural drawdown ecosystems. Long-term *in-situ* monitoring across multi-year water fluctuation cycles is required to accurately evaluate the long-term SOC budget under climate warming, particularly vertical deep-profile SOC leaching and mineralization losses that indoor incubation fails to simulate.

### 4.3 Driving factors regulating Q_10_ variation

The effects of afforestation on SOC mineralization and its Q_10_ were controlled by labile carbon–nitrogen substrate availability, mineral protection, and soil structural stability, representing three hierarchical regulators of soil temperature sensitivity across hydrological gradients (Shen et al., 2026). In the non-flooding zone, afforestation reduced the contents of labile carbon fractions (DOC, MBC, POC) and inorganic nitrogen (**Figure 2**, **3**). The decreased nitrogen availability may force microorganisms to decompose native soil organic matter for nutrient acquisition, triggering positive priming effects, accelerating SOC turnover, and increasing Q_10_ (Blagodatskaya and Kuzyakov, 2013; Lin et al., 2025a). Microbes invest more energy into decomposing recalcitrant native SOC under N limitation, and these stable carbon fractions possess higher activation energy and stronger temperature sensitivity, consistent with global carbon quality frameworks (Zhang et al., 2024a). Although afforestation enhanced soil physicochemical protection and SOC stability in the non-flooding zone (**Figure 4**), the intensified SOC decomposition outweighed the protective effect, ultimately increasing Q_10_ (Ke et al., 2023).

**Figure 5:**
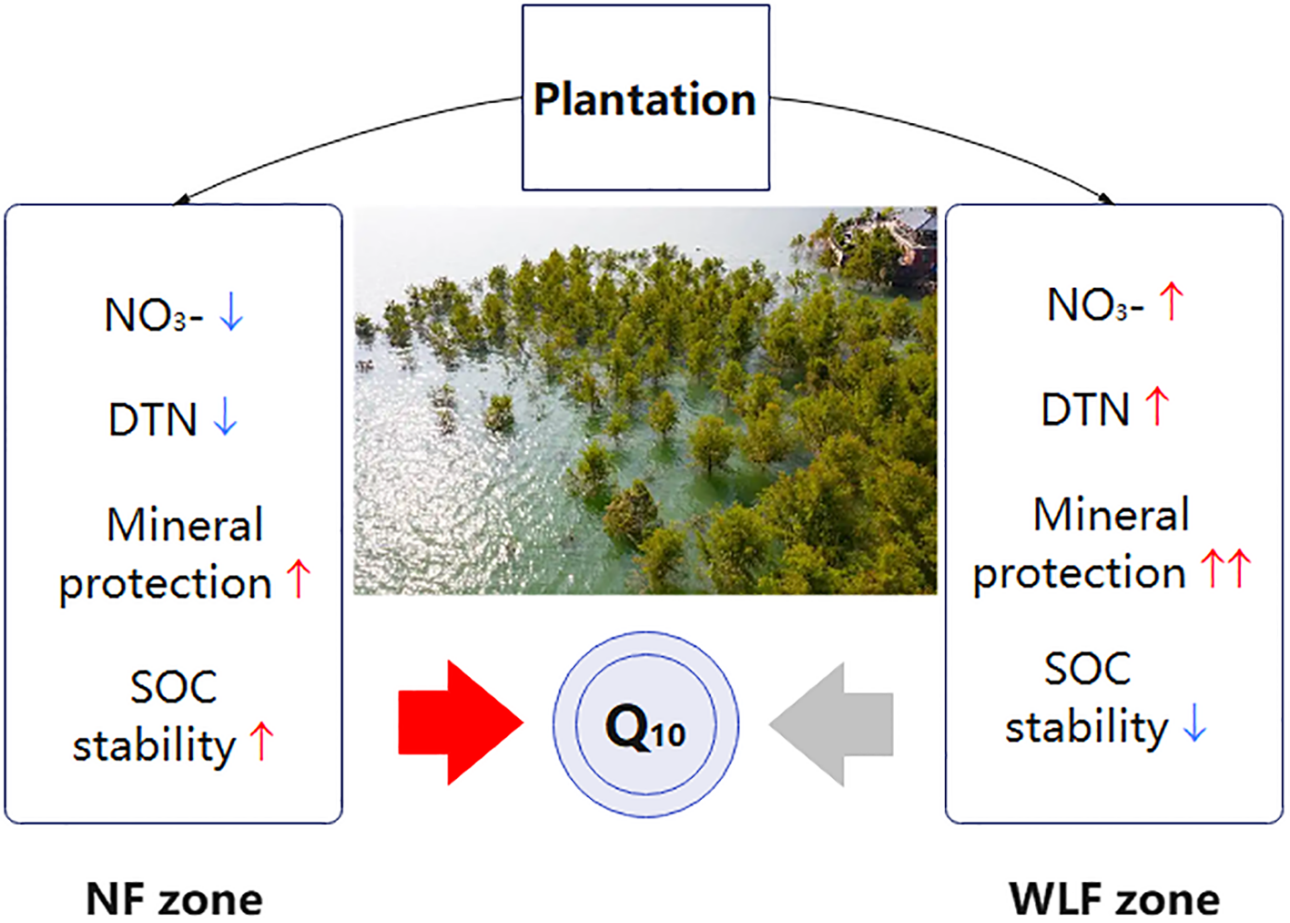
The mechanisms of afforestation effects on the temperature sensitivity of soil organic carbon decomposition in the water level fluctuation zone and non-flooding zone. WLF, water level fluctuation zone; NF, non-flooding zone; Q_10_, temperature sensitivity; DTN, dissolved total nitrogen; NO_3_^-^, nitrate nitrogen; Mineral protection, indicated by the potential of minerals to adsorb SOC, i.e., molar (Fe_d_ + Al_d_)/SOC, exchangeable Ca-to-SOC (Ca_exe_/SOC), and molar (Fe_o_ + Al_o_)/SOC; SOC stability, indicated by rA1635/rA2930 ratio. Red arrows indicate facilitative effects, blue arrows denote inhibitory effects, while gray arrows signify non-significant effects.

In the WLF zone, frequent water-level fluctuations substantially modified SOC stability and thermal responses of SOC decomposition (Zhang et al., 2024b). The water-level fluctuation promoted the potential of iron and aluminum minerals to adsorb SOC during dry periods, and thus reduced the sensitivity of SOC decomposition to temperature changes (Ran et al., 2023; Zhao et al., 2024). Nevertheless, the decreased molecular stability index (rA1635/rA2930) after afforestation indicated weakened SOC structural stability (**Figure 4**), as fresh plant litter inputs elevated alkyl carbon proportions and reduced aromatic recalcitrant compounds, which favored the decomposition of labile SOC fractions (Zhao et al., 2018). The counteracting dual effects of enhanced potential of minerals to adsorb SOC and reduced molecular stability jointly resulted in a relatively stable Q_10_ from afforestation in the WLF zone (**Figure 5**). This bidirectional balance unique to WLF soils explains the divergent afforestation effects on carbon-climate feedback compared with static upland ecosystems, and provides new empirical evidence for parameterizing reservoir WLF zones in Earth system models.

## 5 Conclusions

Hydrological condition is a key regulator of SOC decomposition and its Q_10_ following afforestation. Afforestation exerts hydrology-dependent effects on SOC decomposition rather than a uniform influence across the WLF zone and non-flooding zone. Under non-flooding conditions, plantation overrides the potential of minerals to adsorb SOC and structural stability, stimulating microbial decomposition via altered labile SOC and nitrogen substrate status. This process accelerates SOC mineralization and its temperature dependence. In contrast, periodic hydrological variation reshapes the regulatory pathway of afforestation on SOC decomposition processes. The water-level fluctuation enhanced the potential of minerals to adsorb SOC, offsetting the loss of SOC molecular structural stability. The counterbalancing effects stabilize microbial thermal responses and do not change the Q_10_ of SOC mineralization in fluctuating hydrological habitats. Hydrological heterogeneity further influenced the distribution of soil carbon and nitrogen fractions, highlighting the importance of flooding dynamics in regulating substrate availability and SOC stabilization mechanisms. These findings demonstrate that flooding disturbance mediates the carbon-climate feedback in restored WLF ecosystems and underscores the necessity of incorporating hydrological variability into the evaluation of carbon sequestration potential and climate mitigation benefits of afforestation practices.

## Data Availability

The raw data supporting the conclusions of this article will be made available by the authors, without undue reservation.
